# Editome landscape of CCM-derived endothelial cells

**DOI:** 10.1080/15476286.2022.2091306

**Published:** 2022-06-30

**Authors:** Concetta Scimone, Simona Alibrandi, Luigi Donato, Concetta Alafaci, Antonino Germanò, Sergio L. Vinci, Rosalia D’Angelo, Antonina Sidoti

**Affiliations:** a Department of Biomedical and Dental Sciences and Morphofunctional Imaging, University of Messina, Messina, Italy; b therapies, I.E.ME.S.TDepartment of Biomolecular strategies, genetics, cutting-edge, Palermo, Italy; c Department of Chemical, Biological, Pharmaceutical and Environmental Sciences, University of Messina, Messina, Italy; d Neurosurgery Unit, Department of Biomedical and Dental Sciences and Morphofunctional Imaging, University of Messina, Messina, Italy; e Neuroradiology Unit, Department of Biomedical and Dental Sciences and Morphofunctional Imaging, University of Messina, Messina, Italy

**Keywords:** Cerebral cavernous malformation, editome profile, protease-activated receptor signalling, RNA sequencing analysis, differentially edited genes

## Abstract

By regulating several phases of gene expression, RNA editing modifications contribute to maintaining physiological RNA expression levels. RNA editing dysregulation can affect RNA molecule half-life, coding/noncoding RNA interaction, alternative splicing, and circular RNA biogenesis. Impaired RNA editing has been observed in several pathological conditions, including cancer and Alzheimer’s disease. No data has been published yet on the editome profile of endothelial cells (ECs) isolated from human cerebral cavernous malformation (CCM) lesions. Here, we describe a landscape of editome modifications in sporadic CCM-derived ECs (CCM-ECs) by comparing editing events with those observed in human brain microvascular endothelial cells (HBMECs). With a whole transcriptome-based variant calling pipeline, we identified differential edited genes in CCM-ECs that were enriched in pathways related to angiogenesis, apoptosis and cell survival, inflammation and, in particular, to thrombin signalling mediated by protease-activated receptors and non-canonical Wnt signalling. These pathways, not yet associated to CCM development, could be a novel field for further investigations on CCM molecular mechanisms. Moreover, enrichment analysis of differentially edited miRNAs suggested additional small noncoding transcripts to consider for development of targeted therapies.

## Introduction

Conversions of adenosine (A) to inosine (I) (A-to-I) and cytosine (C) to uracil (U) (C-to-U) are the most frequent post-transcriptional editing modifications occurring in RNA [1], and this mechanism of regulation is known as canonical RNA editing. A-to-I enzymatic deamination is mainly catalysed by a deaminase family of enzymes known as adenosine deaminases acting on RNA (ADARs) that comprises the three members ADAR1 (*ADAR*, 1q21.3, HGNC: 225), ADAR2 (*ADARB1*, 21q22.3, HGNC: 226), and ADAR3 (*ADARB2*, 10p15.3, HGNC: 227) [2,3]. Apolipoprotein B mRNA-editing enzyme, catalytic polypeptide (APOBEC) deaminase family members, instead, are responsible for C-to-U conversion [4]. Chemically, in edited RNA molecules, cellular machinery reads inosine as guanosine (G), mimicking an A-to-G transition in codons, regulatory regions and noncoding RNAs [5,6]. These changes can result in amino acid substitutions, alternative splicing, alteration of secondary structure, perturbation of gene expression regulation, spatial redistribution and decay of edited transcripts. To date, more than 4.5 million A-to-I editing events have been predicted to occur in human tissues and they are not randomly distributed across the genome [7]. In detail, it was shown that short interspersed elements (SINEs), long interspersed elements (LINEs) including Alu elements, and retrotransposons are particularly enriched in editing sites [8,9]. Moreover, editing sites seem to be highly represented both in ion channels and neurotransmitter receptor transcripts, in the central nervous system (CNS) [10]. Interestingly, a recent study demonstrated a close interaction between RNA deamination and N^6^-methyladenosine (m^6^A) abundance. RNA N^6^-methylation is a reversible reaction catalysed by specific methyltransferases and represents an exceptional kind of epigenetic regulation at the RNA level. In particular, it was shown that A-to-I conversion is enhanced following m^6^A depletion, as well as the fact that A-to-I conversion may block further RNA N^6^-methylation [11]. Dysregulation of ADAR family members has been reported in several pathological conditions, such as cancer and neurodegenerative diseases [12]. Moreover, loss of function mutations in *ADAR* genes are linked to an inherited infantile encephalopathy known as Aicardi-Goutières syndrome (AGS) [13]. Following implementation of ‘omic’ technologies, the term ‘editome’ was coined, referring to the whole pool of edited sites within a specific tissue and *ad hoc* databases have been created to collect high-throughput data reporting editing changes in both physiological and pathological conditions [14,15]. In this context, the editome profile of cerebral cavernous malformation (CCM, OMIM *#*116860) endothelial cells (ECs) is still an uncharacterized landscape. CCM is a pathological conditions of brain capillaries affecting up to 0.5% people worldwide. The disease can develop sporadically or be inherited as an autosomal dominant character due to germline mutations at the three genes *CCM1/KRIT1* (7q21-q22, HGNC ID:1573), *CCM2/MGC4607* (7p15-p13, HGNC ID:21708) and *CCM3/PDCD10* (3q26.1, HGNC ID:8761) [16–18]. Following CCM gene loss of function mutations, CCM endothelial cells (CCM-ECs) show defects in both tight and adherens junctions and a reduced number of surrounding pericytes, resulting in a defective and badly organized monolayer endothelium with a consequent increase of blood-brain barrier (BBB) permeability [19]. However, according to the variable expressivity and the incomplete penetrance data collected from patients, as well as the absence of germline mutations in CCM genes in patients affected by the inherited form of the disease, it is well accepted that further genetic factors may contribute to CCM development and progression [20]. In order to better understand the complex molecular cascade triggering pathogenesis of the CCM phenotype, several expression studies have been performed revealing a large amount of both coding and noncoding genes, which are dysregulated in CCM animal models [21–24]. We also recently performed whole transcriptome analysis on ECs isolated from CCM biopsies identifying perturbation of the non-canonical Wnt/planar cell polarity pathway and of the Ca^2+^ ion homoeostasis-related pathways [25]. Thus, starting with transcriptome data, we aim to draft the first editome profile of CCM-ECs. The reason for this study is to identify differential editing events particular to CCM-ECs to increase knowledge on CCM pathogenesis by considering a still unexplored mechanism of gene expression regulation.

## Materials and methods

### Sample collection, processing and genotyping

As previously described [25], CCM-ECs were isolated from 2 biopsies belonging to two patients affected by sporadic CCM harbouring no germline mutations at the three CCM genes (CCM-ECs1 and CCM-ECs2). Cells were genotyped and no somatic mutations were identified at the same loci. Informed consent was obtained for all patients enrolled in the study.

### Human brain microvascular endothelial cells culture

T-25 flasks were coated with Matrigel® Matrix (Corning, New York, NY, U.S.A.) for primary cultures of human brain microvascular endothelial cell (HBMEC, Neuromics®, Edina, MN, USA) growth. ENDO-Basal Media supplied with ENDO-Growth Supplement and 1% penicillin/streptomycin was used. Incubation was conducted at 37°C with 5% CO_2_.

### Whole RNA sequencing

Transcriptome analysis was performed on RNA purified from CCM-ECs_1 and CCM-ECs_2. HBMECs were also processed in order to obtain a control ‘editome’ profile. In detail, 1 μg of total RNA for each reaction was used to obtain paired-end libraries with the TruSeq® Stranded Total RNA Sample Prep Kit with Ribo-Zero H/M/R (Illumina® Inc., San Diego, CA, USA). Following amplification, libraries were run on a NovaSeq 6000 System sequencer (Illumina® Inc., San Diego, CA, USA) using the NovaSeq 6000 SP Reagent Kit (Illumina® Inc., San Diego, CA, USA). For each sample, three biological replicates were considered.

### Raw data pre-processing and RNA editing site detection pipeline

Generated FASTQ data were processed by Trimmomatic (v.0.39) [26] to remove low quality reads (average Phred score <29). Adapters and poli(A) sequences were removed by Cutadapt [27]. Filtered data were mapped against the GRCh38 human reference genome by the HISAT2 aligner [28]. Duplicate reads were removed by the MarkDuplicate tool provided by Picard toolkit (v.2.18.23) (‘Picard Toolkit’. 2019. Broad Institute, GitHub Repository. http://broadinstitute.github.io/picard/; Broad Institute). Subsequently, the recalibration of the aligned reads was performed by the Genome Analysis Toolkit (GATK) (v.4.1.3.0) (https://software.broadinstitute.org/gatk/). For editing site annotation, REDItools scripts were used and results mapped against the REDIportal V2.0 database [29].

### Editing sites classification and filtering criteria

REDItools/REDIportal output consists of both annotated and *de-novo* editing sites. For both groups, editing sites were classified according to the nucleotide substitution as A-to-I, C-to-U and miscellaneous, comprising all unconventional RNA editing events. *De-novo* editing sites were filtered according to the Bonferroni-adjusted p-value and only those showing a p-value < 0.05 were selected for downstream analysis. However, while loci spanned by annotated editing sites are indicated in the REDIportal output file, this is not the case for de-novo sites. Therefore, the Variant Effect Predictor (VEP) tool of the Ensemble Genome Browser (https://www.ensembl.org/Homo_sapiens/Tools/VEP?db=core) was used for annotation of the *de-novo* editing sites as well as for classification, according to functional class [30]. In order to proceed to downstream analysis with the same data format, also the already annotated editing sites were run with the VEP tool. According to VEP prediction, only editing modifications showing ‘HIGH’, ‘MODERATE’ and ‘MODIFIER’ impact on gene expression or function were considered for downstream analysis. Finally, due to their uncertain consequences on RNA molecule fate, miscellaneous editing events were excluded.

### Gene clustering

Edited loci were selected in relation to the impact of the spanning editing sites. These loci were then clustered according to the differential distribution of the editing sites between HBMEC and CCM-EC samples. In detail, three groups of genes were considered. The first was obtained by genes edited in both HBMEC and CCM-EC samples but showing different editing events and frequency. The editing ratio was calculated for each editing site shared by both CCM-EC and HBMEC samples. Frequencies of editing events were calculated by IBM SPSS Statistics 26.0 software [31]. The second cluster comprises genes only edited in CCM-EC samples; the third one includes genes only edited in HBMECs.

### Functional enrichment of differential-edited genes

To identify pathways which genes with different editing sites (DESs) are involved in, enrichment analysis was performed by the FunRich v.3.1.3 tool [32]. In detail, only genes presenting conventional A-to-I and C-to-U editing modifications were functionally enriched according to biological pathways of the Reactome Pathway database [33]. However, the FunRich tool returns annotations including ‘Cellular component’, ‘Molecular function’ and ‘Biological process’ of the Gene Ontology knowledgebase, protein domain, site of expression, transcription factor binding, clinical phenotype, and somatic mutations collected in the COSMIC database. False discovery rate is calculated by the hypergeometric test and adjusted by both the Bonferroni and the Benjamini–Hochberg corrections. Only results showing a Bonferroni-adjusted p-value < 0.05 were considered. Moreover, the FunRich tool also allows functional enrichment of miRNAs. Therefore, a fourth dataset containing all differentially edited miRNAs in CCM-ECs was created. Similarly, in this case, only ‘Biological pathways’ showing a Bonferroni-adjusted p-value < 0.05 were considered.

## Results

### Total RNA sequencing and raw data pre-processing

A mean of 57,393,366.44 total reads (8.67 Gbases) were outputted from the three replicas of both CCM-ECs and HBMECs (SM1). Phred quality score ≥29 was shown by 97.57% of the total generated reads that were filtered and considered for downstream analysis. Regarding mapping, an average of 81,781,921 reads were mapped against the GRCh38 human reference genome and, of these, 65.38% uniquely mapped. REDItools annotation allowed to detect both annotated and *de-novo* editing sites in HBMECs and CCM-ECs (SM2) and results are summarized in Table 1.

**Table 1.** **Editing sites in HBMECs and CCM-ECs**. For each sample, the total number of both annotated and *de-novo* editing sites is reported. Annotated sites are divided according to the deamination reaction (A-to-I and C-to-U). ‘Miscellaneous’ group comprises all unconventional editing modifications. The same classification is made for *de-novo* editing sites showing a Bonferroni-adjusted p value < 0.05.

Editing sites	HBMECs	CCM-ECs1	CCM-ECs2
Annotated	22,859	136,623	38,274
	11,273 A-to-I	69,468 A-to-I	18,709 A-to-I
	0 C-to-U	3 C-to-U	2 C-to-U
	11,586 Miscellaneous	67,152 Miscellaneous	19,564 Miscellaneous
*De-novo* (total)	21,964	364,413	121,750
*De-novo* (Bonferroni-adjusted pValue < 0.05)	1,576	35,140	10,347
	369 A-to-I	11,017 A-to-I	1,985 A-to-I
	249 C-to-U	3,330 C-to-U	1,778 C-to-U
	958 Miscellaneous	20,793 Miscellaneous	6,584 Miscellaneous

### Large number of differentially edited sites (DESs) between CCM-ECs and HBMECs

**Figure 1.**
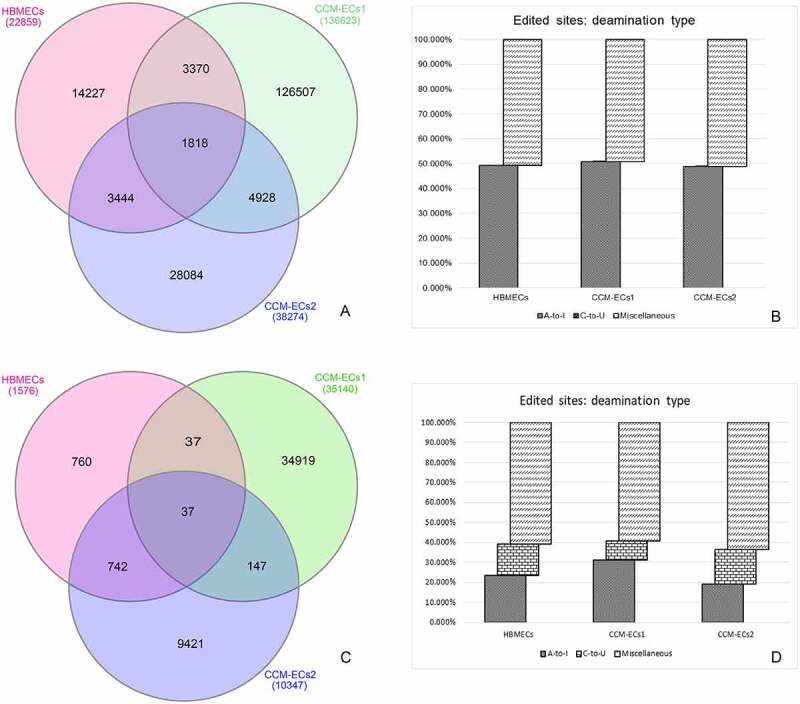
**Edited sites distribution in CCM-ECs and in HBMECs**. Venn diagrams show annotated (a) and *de-novo* (c) DESs in the two samples (CCM-ECs1 and CCM-ECs2) and in HBMECs. Bar charts (b, d) represent the editing site distribution, according to the nucleotide modification (A-to-I, C-to-U, miscellaneous). Miscellaneous group comprises all non-canonical editing modifications. A much lower percentage of C-to-U deamination events was detected in annotated editing sites (b) when compared to the *de-novo* ones (d). Venn diagrams were obtained by the InteractiVenn tool [69].

Comparison of editing profiles between CCM-ECs and HBMECs highlighted a huge number of both annotated and *de-novo* DESs (SM3). In detail, <8% of all HBMEC annotated editing sites were shared with both CCM-EC samples (Figure 1(a)). A-to-I modification represented approximately half of all annotated editing events. In contrast, C-to-U deamination was infrequent (Figure 1(b)). Regarding *de-novo* editing events, most of the editing sites are unique for each sample (Figure 1(c)) and non-canonical modifications were the most represented (Figure 1(d)). However, due to their still uncertain biological consequences on transcript fate, they were not considered for downstream analysis. As shown, the number of *de-novo* editing events outputted by the REDItools is considerably larger in CCM-ECs rather than in HBMECs (Table 1). Interestingly, 4,928 and 147 of annotated and *de-novo* events, respectively, are common to both CCM-ECs1 and CCM-ECs2.

### Distribution of RNA editing sites and functional classification

**Figure 2.**
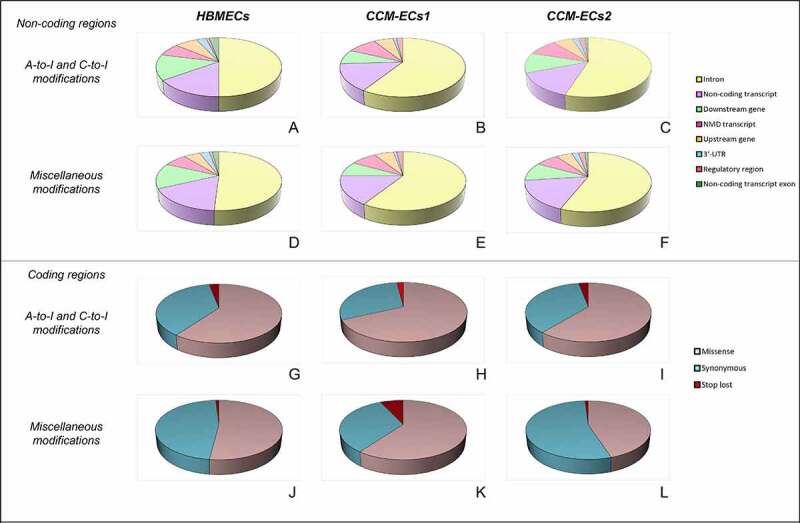
**Genomic distribution of annotated editing sites**. In noncoding regions (a-f), editing sites were mainly mapped within introns and in noncoding transcripts. Modifications are clustered according to the nucleotide deamination. In detail, the canonical editing modifications A-to-I and C-to-U are grouped in the same charts (a-c), while the non-canonical ones are comprised within the miscellaneous group (d-f). Distribution across coding sequences is shown in panels g-l. Canonical editing modifications mostly result in missense variants (g-i).

RNA editing sites are distributed both on coding and noncoding genome regions. In noncoding regions, annotated editing sites mostly span along introns (Figure 2(a-f)). Editing modifications occurring in sequences involved in regulation of gene expression are represented in almost the same percentages, in the three samples. Moreover, this distribution is homogeneous between canonical A-to-I and C-to-U (Figure 2(a-c)) and non-canonical editing modifications (Figure 2(d-f)). Deamination reactions affecting coding regions, indeed, mainly lead to missense mutations (Figure 2(g-i)). Interestingly, modifications result in stop codon loss in about 3% of genes edited in coding sequence (SM4a-d).

**Figure 3.**
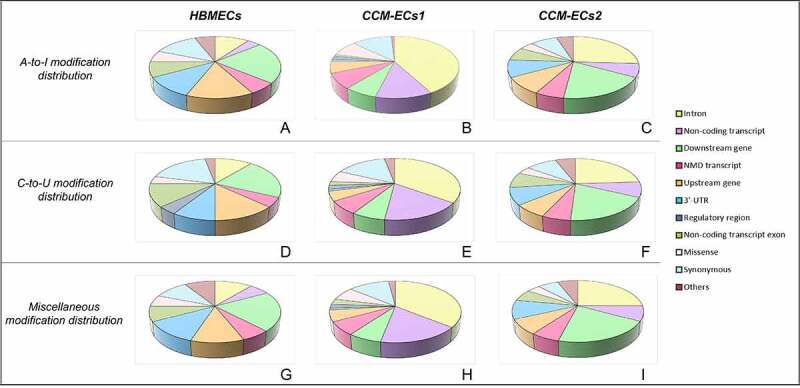
**Genomic distribution of *de-novo* editing sites**. *De-novo* editing sites were annotated by the Variant Eeffect Predictor tool of the Ensembl Genome browser. Editing sites mainly overlap with noncoding regions. Modifications are grouped in relation to deamination reaction as A-to-I (a-c), C-to-U (d-f), miscellaneous (g-i).

Annotation of the *de-novo* editing sites by the VEP tool of the Ensembl Genome browser for HMBEC and CCM-EC samples revealed that only <10% of all detected sites were really novel substitutions (Table 2). Most of them, indeed, are known variants but not yet related to editing events, requiring further validation (SM5). In CCM-ECs1, the percentage of novel variants is greater probably due to the larger output generated by sequencing run. Overall, the *de-novo* editing sites mainly occur within noncoding regions (Figure 3).

**Table 2.** **Annotation of the de-novo editing sites**. The table summarizes results of VEP annotations of the *de-novo* editing sites. For each sample, editing events were divided according to the enzymatic modification. For each group, the number of processed variants is reported. This value refers to the editing events outputted by the REDItools and showing a Bonferroni-adjusted p value < 0.05. Most of these are already annotated as shown by the ratio between novel and existing variants (Novel(%)/existing(%) variants, discussed in the text). The number of edited genes, transcripts and regulatory regions is also indicated.

Sample	Editing modification	Variant processed	Ratio (%)	Overlapped genes	Overlapped transcripts	Overlapped regulatory features
HBMECs	A-to-I	369	30 (8.1)/339 (91.9)	489	2973	126
	C-to-U	249	26 (10.4)/223 (89.6)	358	2261	100
	Miscellaneous	958	101 (10.5)/857 (89.5)	1004	6192	317
CCM-ECs1	A-to-I	11007	6456 (58.7)/4551 (41.3)	5811	32,393	1206
	C-to-U	3320	202 (6.1)/3118 (93.9)	2666	13,646	504
	Miscellaneous	20,679	7403 (35.8)/13,276 (64.2)	9456	49,960	2433
CCM-ECs2	A-to-I	1985	47 (2.4)/1938 (97.6)	2200	13,401	631
	C-to-U	1778	24 (1.3)/1754 (98.7)	1983	12,430	580
	Miscellaneous	6584	145 (2.2)/6439 (97.8)	4816	30,257	1731

### Increased number of editing events in CCM-ECs

**Figure 4.**
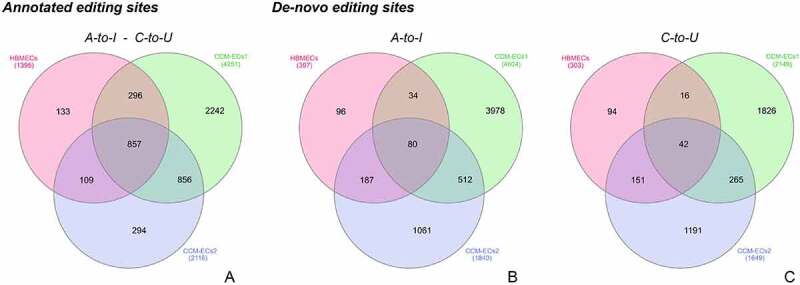
**Distribution of edited genes in CCM-ECs and in HBMECs**. Venn diagram a refers to genes overlapped by annotated A-to-I and C-to-U editing sites, while in b and c panels are grouped genes edited by *de-novo* modifications. Genes are divided in relation to the nucleotide deamination.

Comparison between HBMECs and CCM-ECs highlighted that most genes were differentially edited in CCM-ECs. Among edited genes, 857 undergoing canonical editing (A-to-I and C-to-U) were shared by control cells and both CCM-ECs (Figure 4(a)). Moreover, only 80 and 42 loci are involved in novel A-to-I and C-to-U editing events in both HBMECs and CCM-ECs (Figure 4(b,c)) (SM6). Following the merging of loci spanned by both annotated and *de-novo* editing sites, duplicate genes were discarded. In total, 858 loci remained, suggesting that the same loci are affected by both annotated and *de-novo* editing events.

In genes overlapped by annotated editing sites, A-to-I modification is the most frequent. Interestingly, C-to-U deamination only occurred within 3 and 2 genes of the CCM-ECs1 and CCM-ECs2, respectively, while it was not observed in HBMECs. Regarding *de-novo* editing sites, non-canonical events are the most represented (Table 3).

**Table 3.** **Genes overlapped by edited sites in HBMECs and CCM-ECs**. Genes overlapped by both annotated and *de-novo* editing sites are considered. The total gene number is divided according to the editing modification.

Overlapped genes	HBMECs	CCM-ECs1	CCM-ECs2
Annotated editing sites	1,395 A-to-I	4,251 A-to-I	2,116 A-to-I
	0 C-to-U	3 C-to-U	2 C-to-U
	1,389 Miscellaneous	4,335 Miscellaneous	2,185 Miscellaneous
De novo (Bonferroni-adjusted p value < 0.05) editing sites	397 A-to-I	4,604 A-to-I	1,840 A-to-I
	303 C-to-U	2,149 C-to-U	1,649 C-to-U
	809 Miscellaneous	7,254 Miscellaneous	3,942 Miscellaneous

**Figure 5.**
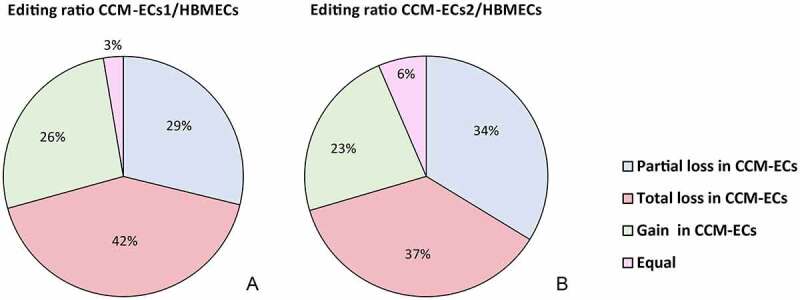
**Differential editing between CCM-ECs and HBMECs**. The pie charts show differences in editing frequency between HBMECs and CCM-ECs1 (a) and CCM-ECs2 (b). As discussed in the text, most of editing sites are totally or partially lost in CCM-ECs, when compared to HBMECs. Only 3% and 6% of editing sites show the same frequency between CCM-ECs1 and CCM-ECs2, respectively, and HBMECs.

For each gene edited in both HBMECs and CCM-ECs, editing ratio was considered in order to calculate differences of editing rate between samples and control. In total, 4,499 editing sites were considered, distributed across 1,315 coding (85.9%), noncoding (13.6%), and mitochondrial (0.5%) genes (SM7). As shown in Figure 5, according to editing ratio values, in CCM-ECs >37% and 29% of editing sites are totally and partially lost, respectively. In contrast, 26% and 23% of editing events were increased in CCM-ECs1 and CCM-ECs2, respectively.

**Figure 6.**
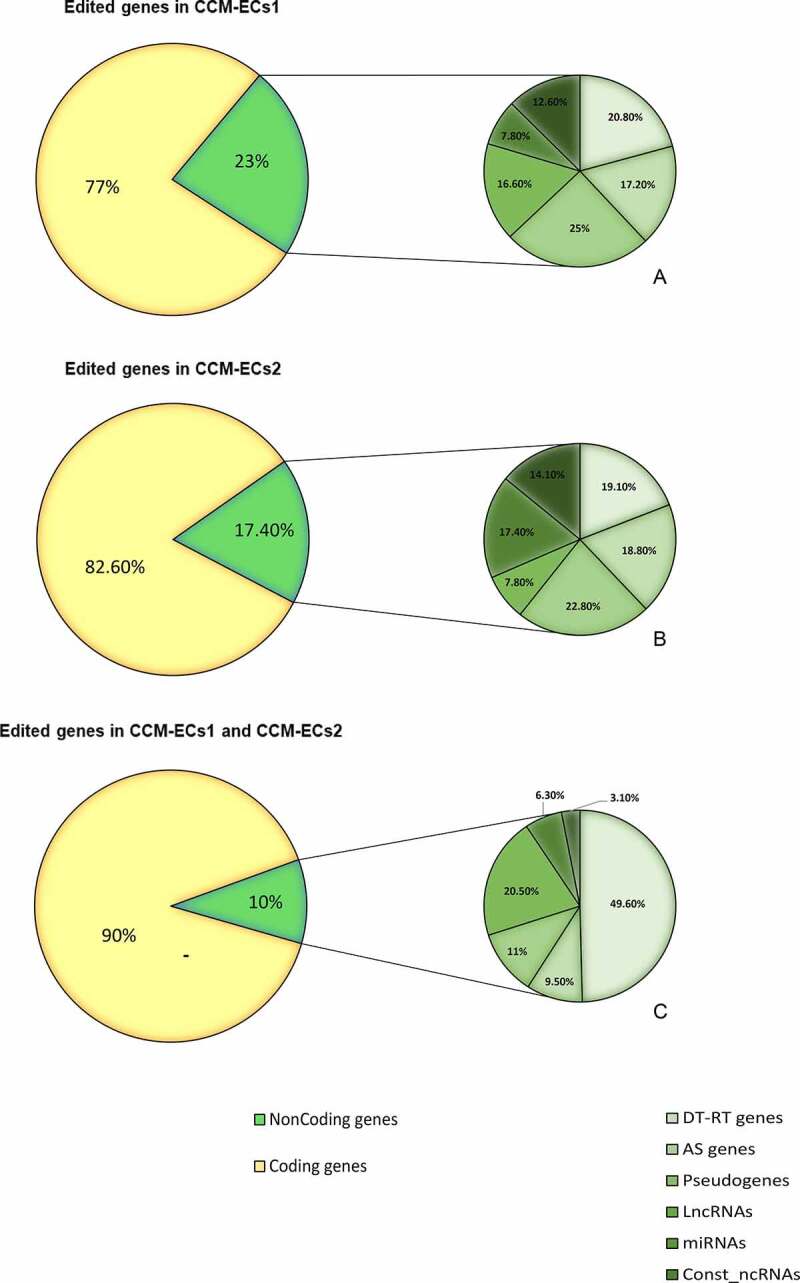
**Distribution of coding/noncoding loci spanned by editing sites**. Editing sites mostly occur in coding genes both in CCM-ECs1 (a), in CCM-ECs2 (b) and in genes edited in both samples (c). The smallest pies represent distribution of editing sites in noncoding regions. DT: divergent transcript; RT: readthrough; AS: antisense; Lnc: long noncoding; Const_nc: constitutive noncoding.

However, a large number of genes were only edited in the two CCM-EC samples (Figure 4) (SM6). In detail, following removal of duplicate genes covered by both annotated and *de-novo* editing sites, 6,430 and 2,246 loci were uniquely edited in CCM-ECs1 and CCM-ECs2, respectively. Moreover, 1,412 loci were edited in both samples. As shown in Figure 6, most of them are coding genes. Transcribed pseudogenes represented the most frequent class of noncoding genes, edited in both CCM-ECs1 and CCM-ECs2, followed by divergent and readthrough transcripts.

### Editing imbalance in genes controlling angiogenesis and inflammation

Following VEP annotations, functional enrichment was performed for genes spanned by high, moderate and modifier A-to-I and C-to-U editing events (Table 4) (SM4-5). Differentially edited genes (DEGs) were clustered in three groups. In detail, the first cluster comprised genes edited in both HBMECs and in CCM-ECs but showing different editing sites or different editing frequency. In total, 1,315 genes were given as input to the FunRich tool. Of these, 164 were not recognized as they were noncoding transcripts and pseudogenes, while the remaining were clustered in 1,000 ‘Biological pathways’ (SM8a) and, of these, 40 were considered (Bonferroni-adjusted p-value < 0.05). These pathways are involved in integrin signalling transduction, angiogenesis, inflammation, apoptosis, cell metabolism, and protease-activated receptors (PARs) signalling. In detail, 151 genes were enriched in these pathways and most of them are common to all biological processes considered.

**Table 4.** **Editing site distribution according to impact on gene structure**. For each sample, the number of editing events is reported and they are divided in relation to both the enzymatic modification (A-to-I, C-to-U) and the impact weight (high, moderate, modifier).

	HBMECs		CCM-ECs1		CCM-ECs2	
	A-to-I	C-to-U	A-to-I	C-to-U	A-to-I	C-to-U
Annotated_High	14	0	13	0	16	0
Annotated_Moderate	36	0	79	0	44	0
Annotated_Modifier	31,546	0	178,198	6	53,129	3
De-novo_High	2	0	4	0	4	13
De-novo_Moderate	65	30	23	14	296	248
De-novo_Modifier	1,068	709	26,352	6,672	5,774	5,147
Total editing sites	33,470		211,361		64,674	
Covered genes	2,288		8,359		4,762	

The second cluster included only genes edited in CCM-EC samples. This gene set was made up of 8,033 genes. However, a large number of loci were rDNA, LINC, RNU, SNOR genes, regulatory RNAs and uncharacterized loci reducing the number of recognized entries to 6,005. These were clustered in 1,417 ‘Biological pathways’, according to the Reactome annotation terms. However, only 41 of them were selected (Bonferroni-adjusted p-value < 0.05) (SM8b). Also in this case, enriched pathways were related to extra-cellular matrix (ECM) signalling transduction, angiogenesis, inflammation, cell metabolism, and protease-activated receptors (PARs) signalling. In total, 621 genes were clustered in these pathways (Table 5). However, an additional enriched pathway showed ‘Regulation of CDC42 activity’, with 305 genes clustered. Of these, 36 were not shared with the other pathways. In agreement with VEP prediction, *de-novo* high impact editing modifications occurred in *AIFM1* (Xq26.1, HGNC Id: 8768) in CCM-ECs1 and in *ARHGAP26* (5q31.3, HGNC Id: 17,073), *CDK1* (10q21.2, HGNG Id: 10q21.2) and *SPP1* (4q22.1, HGNC Id: 11,255) in CCM-ECs2. AIFM1 was clustered in the ‘Sphingosine 1-phosphate (S1P) pathway’ and in the ‘TRAIL signalling pathway’, *ARHGAP26* and *SPP1* in all pathways with the exception of ‘Regulation of CDC42 activity’, while *CDK1* was identified in all enriched pathways.

**Table 5.** **Enriched pathways for differential edited genes (DEGs)**. As discussed in the text, both DEGs of the first cluster (here in the table ‘HBMECs and CCM-ECs’) and of the second cluster (in the table ‘CCM-ECs’) were enriched in the same pathways. However, the number of clustered genes is largely increased in CCM-ECs samples. *Pathway only enriched in CCM-ECs.

			HBMECs and CCM-ECs		CCM-ECs	
Macropathway	Biological pathways	Gene in the background dataset	Gene of the dataset	P-value (Bonferroni correction)	Gene of the dataset	P-value (Bonferroni correction)
Integrin signalling	Syndecan-1-mediated signalling events	1297	135	0.000103169	504	0.011101059
	Proteoglycan syndecan-mediated signalling events	1342	138	0.00014797	523	0.004503906
	Nectin adhesion pathway	1292	134	0.000148989	501	0.015908641
	Glypican 1 network	1296	132	0.000593664	502	0.017978423
	Beta1 integrin cell surface interactions	1348	136	0.000636339	522	0.011458661
	Alpha9 beta1 integrin signalling events	1302	132	0.000782412	504	0.018666533
	Integrin family cell surface interactions	1375	137	0.001204981	533	0.007540158
	Glypican pathway	1335	133	0.00194036	513	0.039913015
	Signalling events mediated by focal adhesion kinase	1285	132	0.000354597	499	0.013791016
Angiogenesis	VEGF and VEGFR signalling network	1301	132	0.00074741	507	0.006922936
	Signalling events mediated by VEGFR1 and VEGFR2	1293	132	0.000516438	502	0.013169074
	PDGF receptor signalling network	1290	132	0.000448859	505	0.017163105
	Endothelins	1304	133	0.000479927	500	0.01730961
	PDGFR-beta signalling pathway	1285	132	0.000354597	499	0.013791016
Thrombin signalling	PAR1-mediated thrombin signalling events	1296	133	0.000329645	503	0.013427222
	Thrombin/protease-activated receptor (PAR) pathway	1297	133	0.000345611	503	0.014900849
	Urokinase-type plasminogen activator (uPA) and uPAR-mediated signalling	1285	132	0.000354597	499	0.013791016
Inflammation	IL3-mediated signalling events	1292	133	0.000272554	500	0.021258521
	IFN-gamma pathway	1293	133	0.000285868	504	0.007250779
	IL5-mediated signalling events	1289	132	0.000428274	501	0.011626294
	GMCSF-mediated signalling events	1289	132	0.000428274	499	0.020864765
EGFR signalling	ErbB1 downstream signalling	1285	132	0.000354597	499	0.013791016
	ErbB receptor signalling network	1308	132	0.00102756	509	0.008023272
	EGFR-dependent Endothelin signalling events	1286	133	0.000204297	499	0.015308061
	Internalization of ErbB1	1285	132	0.000354597	499	0.013791016
	EGF receptor (ErbB1) signalling pathway	1285	132	0.000354597	499	0.013791016
Apoptosis	TRAIL signalling pathway	1325	135	0.000395007	514	0.010928798
Cell metabolism	Signalling events mediated by Hepatocyte Growth Factor Receptor (c-Met)	1290	133	0.000247683	501	0.012914718
	Insulin Pathway	1285	132	0.000354597	499	0.013791016
	Class I PI3K signalling events	1285	132	0.000354597	499	0.013791016
	Class I PI3K signalling events mediated by Akt	1285	132	0.000354597	499	0.013791016
	mTOR signalling pathway	1285	132	0.000354597	499	0.013791016
	S1P1 pathway	1285	132	0.000354597	499	0.013791016
	LKB1 signalling events	1305	133	0.000502776	508	0.007863929
	Sphingosine 1-phosphate (S1P) pathway	1308	133	0.00057772	509	0.008023272
	Plasma membrane oestrogen receptor signalling	1298	132	0.000651142	506	0.0067828
	IGF1 pathway	1288	132	0.000408592	502	0.007750019
	Arf6 trafficking events	1285	132	0.000354597	499	0.013791016
	Arf6 downstream pathway	1285	132	0.000354597	499	0.013791016
	Arf6 signalling events	1285	132	0.000354597	499	0.013791016
Cdc42 ignalling*	Regulation of CDC42 activity	768	Not significant		305	0.030405136

Finally, the third cluster comprising genes only edited in HMBECs was functionally enriched. It counted 235 both coding and noncoding genes that did not undergo editing deamination in CCM-ECs samples. The FunRich tool recognized 169 genes that were clustered in 386 biological pathways. However, none of these showed statistical significance, according to both Bonferroni and Benjamini-Hochberg-adjusted p-values (SM8c). These data suggest that these editing events are very likely physiological in endothelial cells of the BBB.

### Differentially edited miRNAs regulate genes involved in CCM pathogenesis

**Figure 7.**
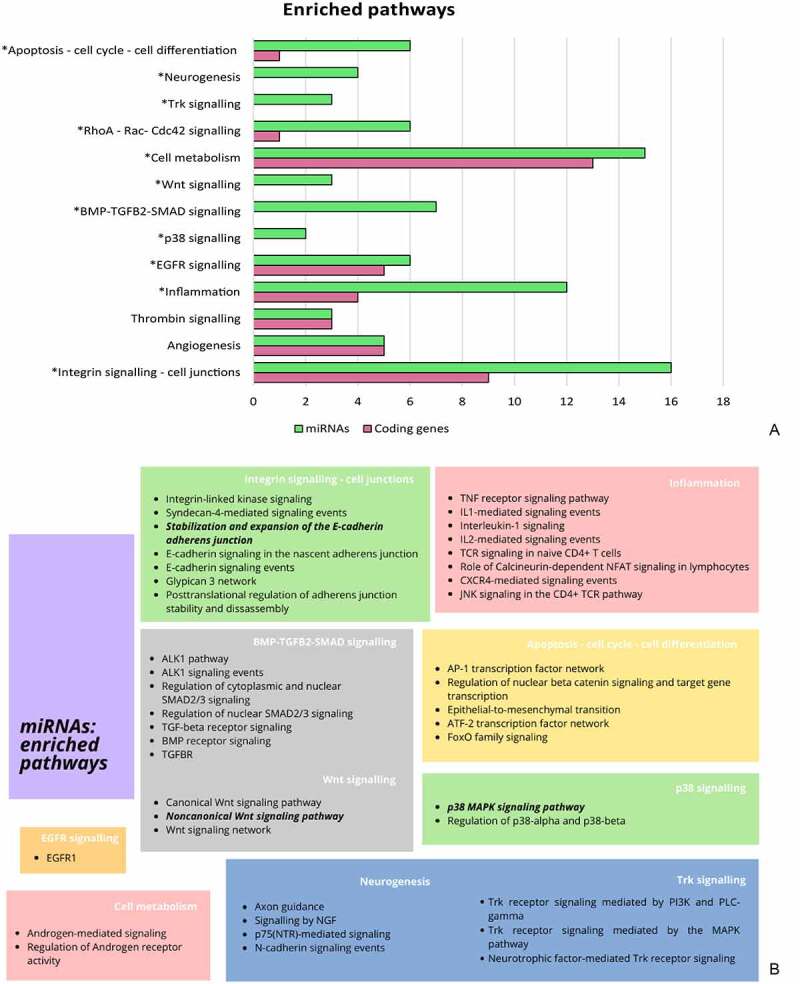
**Pathways enriched by genes targeted by differentially edited miRNAs in CCM-ECs**. Comparison between number of pathways enriched by differential edited genes and genes targeted by differentially edited miRNAs (a) highlights as differential editing in miRNAs affects molecular cascades involved in CCM pathogenesis. With few exceptions, the number of enriched biological processes is larger for miRNA group, for each macropathway (*). These further pathways are elucidated in the b panel.

In total, 207 miRNAs were given as input and 185 were recognized by the FunRich tool. In relation to their targets, differentially edited miRNAs were enriched in 494 biological pathways and, of these, 88 were statistically significant (SM8d). Most of the enriched pathways overlapped with those obtained by functional clustering of DEGs (Figure 7(a)). However, among signalling events not previously detected there were the TGFBR, the p38 and the Wnt pathways, the ‘stabilization of E-cadherin at adherens junctions’, and the neurogenesis-related signalling (Figure 7(b)). Perturbation of these pathways has been described in CCM pathogenesis, as the three CCM proteins are part of them. In endothelial cells of the BBB, indeed, all three CCM proteins co-localize at the adherens junctions to keep them intact [34]. CCM2 is a scaffold protein acting as a negative regulator of the p38 MAPK signalling pathway [35], while KRIT1 modulates the Wnt/β-catenin and the BMP-TGFB cascades [36]. However, our analysis also highlighted the enrichment of the ‘noncanonical Wnt signalling pathway’, *β*-catenin independent. Taken together, our results confirm dysregulation of these molecular cascades in CCM pathological endothelial cells following impairment of miRNAs/target gene interaction, due to differential editing modifications occurring in miRNAs.

### Editing modifications in CCM genes

Finally, we wanted to asses editing modifications which occurred in CCM genes. In detail, 6 and 10 non-canonical T-to-C editing events were annotated in the *KRIT1* gene in HBMECs and in CCM-ECs1, respectively. Regarding the *CCM2* gene, this was largely edited in CCM-ECs1 showing 66 A-to-I, one C-to-U and one T-to-C editing events. Of these, 59 span the noncoding transcript ENST00000461377.5 while 7 occur in intronic regions of the canonical transcript (ENST00000258781.11). Eleven A-to-I modifications were, instead, annotated in CCM-ECs2 in the *CCM2* gene. No editing events were identified in the *PDCD10* locus (SM4-SM5).

## Discussion

RNA editing events are key modulators of RNA molecule balance within cells. Ensuing modifications result in regulation of transcript biological activity and they often differ among cell types contributing to increased variability of RNA pools in tissues. In the same way, editome profiles can show differences in the same cytotype according to physiological or pathological conditions. Although editome profiling is largely characterized in cancer tissues and in neurodegeneration diseases, knowledge on RNA editing perturbation in CCM-ECs is still missing. Given that the pathogenesis of sporadic CCM is mostly unknown, we wanted to investigate the CCM-EC editome profile, obtained by whole RNA sequencing data, and compare it with the HBMEC profile. According to the annotation performed against the REDIportal, the first observation is related to the absence in the database of the human BBB EC editome profile: available data were obtained from aorta, tibial artery and coronary human samples. From HBMEC whole transcriptome sequencing, we identified 22,859 annotated and 1,576 *de-novo* editing sites in HBMECs, suggesting that these events may be particular to BBB ECs. Moreover, about 60% of genes edited in HBMECs were also modified in CCM-ECs. However, editing ratio values calculated for each editing site overlapping genes differentially edited in both HBMEC and CCM-EC samples showed that > 37% of them are totally lost in CCM-ECs, while about 30% are partially lost. Most of these genes encode for proteins involved in ECM remodelling. In contrast, an increase of editing events was observed in < 26% of loci in CCM-ECs. Among these, the noncoding ENST00000461377.5 transcript of the *CCM2* gene was highly modified. This transcript was shown to use an alternate promoter and 5’ exon and the start codon is missing. For these reasons, to date it is classified as a noncoding RNA with unknown functions; however, a coding role is not excluded [37]. As highlighted by enrichment analysis, DEGs contribute to regulation of angiogenetic processes and, in particular, to integrin transduction signalling. Involvement of β1-integrin in CCM progression is widely confirmed and it is well known that CCM proteins contribute to cell adhesion stabilization and cytoskeleton dynamics regulation [38]. Therefore, these data suggest that dysregulation of further genes involved in the same cascade can play a role in CCM disease pathogenesis and, in this context, detected editing modifications can affect half-life and activity of differential edited transcripts. Another pathway related to cytoskeleton organization and cell polarity maintenance involves the cell division control protein 42 homolog (Cdc42) protein. Among genes differentially edited in CCM-ECs, 305 were clustered in the ‘Regulation of CDC42 activity’ pathway. It was demonstrated that *CDC42* depletion in endothelial cells results in CCM-like phenotype onset and this mechanism involves the RhoA-ROCK, MEKK3-MEK5-ERK5-KLF2/4 cascade [19,39]. Taken together, our data match well to what was reported in literature suggesting that also editing imbalance can contribute to sporadic CCM development. Moreover, we observed a high percentage of genes clustered in inflammation-related pathways. Increased immune cell infiltration and pro-inflammatory cytokine synthesis characterize CCM maturation but not lesion formation [22]. In particular, the IFN-γ pathway was enriched in CCM-ECs, according to DEG clustering. IFN-γ was recently designated as a prognostic factor in CCM disease as plasma levels were associated to a more aggressive clinical course [40]. Moreover, in our samples, interleukin 3 (IL-3), interleukin 5 (IL-5) and granulocyte-macrophage colony-stimulating factor (GM-CSF) signalling pathways were highly enriched. These cytokines act on endothelial cells enhancing growth and migration [41]. Interestingly, IL-3 acts as a pro-angiogenetic factor [42] and it was shown to be released not only by immune T-cells but also by non-immune cells of the neurovascular unit, such as microglia and astrocytes, targeting endothelial cells that express the receptor IL-3Rα. Activation of IL-3 Rα results in complement-5a (C5a) cascade amplification [43]. We previously showed that the same CCM-ECs1 and CCM-ECs2 over-express C5a receptors leading to neuroinflammation and increased BBB permeability [25]. In contrast, the effect of IL-5 on angiogenesis is still controversial [44,45]. However, beyond this evidence that further supports literature data, enrichment analysis revealed that more than 500 DEGs in CCM-ECs were clustered in pathways related to thrombin signalling. Thrombin is a serine-protease largely known for its role in the coagulation cascade, acting through proteolytic cleavage on fibrinogen. In recent years, several studies have demonstrated that coagulation proteases can also act on different substrates, including the protease-activated receptor (PAR) family members [46]. PARs comprise four members (PAR1-4) and, among these, PAR1 is constitutively expressed on endothelial and glial cells in the central nervous system. Through proteolytic cleavage, thrombin activates PAR1 on these cells triggering both proangiogenic and proinflammatory cascades. In detail, PAR1 activation leads to matrix metallopeptidases (MMP) activation and shear stress fibre formation resulting in increased BBB permeability [47]. Following functional clustering analysis, the ‘PAR1-mediated thrombin signalling events’ pathway was highly enriched in DEGs, suggesting a possible role of thrombin imbalance and PAR1 signalling amplification in CCM development. Although there is no still evidence of this hypothesis, it was shown that thrombin acts on brain pericytes increasing Akt and ERK1/2 phosphorylation and, then, *MMP-9* release [48]. *MMP-9* over-expression was observed in CCM surgical specimens, following lesion bleeding [49], and protein level also increased in peripheral blood of CCM patients who developed seizures [50]. According to these data, we think that thrombin imbalance could contribute to progression of CCM lesions, by enhancing disruption of the BBB tight junction, mediated by MMP9 [51], and we suggest that this may be a valid field for further investigations.

### ‘High impact’ editing modifications in angiogenetic and inflammatory genes

We focused on genes affected by editing modifications that were predicted as ‘high impact’ on transcript biological activity. These genes are *AIFM1* in CCM-ECs1 and *ARHGAP26, CDK1* and *SPP1* in CCM-ECs2.

*AIFM1* encodes for the apoptosis-inducing factor mitochondria associated 1, a NADH oxidoreductase with proapoptotic function. In response to apoptotic stimuli, it induces mitochondria to release cytochrome c and caspase-9 and moves from the mitochondrial intermembrane space to the nucleus, where it enhances chromosome condensation and fragmentation [52]. Apoptosis enhancement following AIFM1 translocation was observed in cerebral endothelial cells after ischaemia-reperfusion [53]. In our CCM-ECs1, the A-to-I modification occurs within a noncanonical transcript (Ensembl Id: ENST00000527892.5 – AIFM1-006) that encodes for a 43 amino acid protein but that usually undergoes nonsense-mediated decay (NMD). However, this editing modification was annotated as the rs1139851 that results in stop codon loss (p.Ter44ArgextTer5), leading to a more stable transcript and, then, to a longer protein. Biological functions of both the transcript and the protein have not yet been elucidated.

Rho GTPase Activating Protein 26 is the protein product of the *ARHGAP26* gene. It regulates activity of the GTP binding proteins RhoA and Cdc42, by binding focal adhesion kinase (FAK). FAK acts as a bridge between extracellular space and actin-cytoskeleton, by modulating integrin signalling [54]. The C-to-U modification (rs258819) detected in the ENST00000425417.2 – ARHGAP26-207 transcript was predicted as a splice donor variant (c.198 + 2C > T).

Secreted phosphoprotein 1, encoded by the *SPP1* gene, which acts as a cytokine that stimulates IFN-γ and IL-12 synthesis, is also involved in FAK signalling. Despite its pro-inflammatory activity, in brain ischaemic areas it was shown to promote astrocyte process extension contributing to neurovascular unit repair [55]. Also, in this case, the C-to-U modification (rs11728697) detected in the CCM-ECs2 sample was predicted to result in a splice donor site (n.320 + 2C > T) in the processed noncoding transcript ENST00000681973.1 mapped.

No data are available on the C-to-U editing modification identified in the *CDK1* gene, encoding for cyclin-dependent kinase 1. It was mapped in the canonical transcript ENST00000395284.8 – CDK1-203. According to annotation data, the modification affects the last codon of the protein, but no consequences were predicted. CDK1 is a serine/threonine kinase, a subunit of the M-phase promoting factor (MPF), essential for G1/S and G2/M phase transitions of the eukaryotic cell cycle [56]. In brain endothelial cells, under oxidative stress conditions, it was shown to promote a slowdown in the S-to-G2-to-M transition in order to encourage DNA repair [57].

### Differential editing events in miRNAs targeting CCM-related genes

Finally, we focused on edited miRNAs. Enrichment analysis highlighted that several target genes of differentially edited miRNAs are related to pathways involved in CCM onset, such as the p38/MAPK cascade [35], E-cadherin signalling [34], inflammation [58] and integrin transduction signalling [38]. Also, in this case, the ‘PAR1-mediated thrombin signalling events’ pathway was greatly enriched, further supporting data obtained from the functional clustering of DEGs. Interestingly, another important finding regarded the ‘noncanonical Wnt signalling pathway’ in which several genes targeted by differential edited miRNAs were clustered. The noncanonical (β-catenin independent) Wnt pathway is triggered by the Wnt5a ligand of frizzled seven transmembrane receptors (FZD). At the BBB, pericytes secrete Wnt5a that acts on endothelial cells driving their migration [59]. This pathway is mediated by other co-receptors, such as the LDL receptor-related protein (LRP) family members, ROR1 and ROR2, and is known as the Wnt/planar cell polarity pathway as it is involved in cell polarization. In HBMECs, most cellular processes controlled by the pathway include tight junction maintenance [60], cytoskeleton organization and primary cilia disassembly following fluid shear stress stimuli [61]. Fluid shear stress was recently proposed as contributing to CCM signalling activation [62]. In addition, in a previous expression study, for the first time, we showed a high rate of dysregulated genes that are involved in the Wnt/planar cell polarity pathway [25]. Taken together, these data contribute to further consideration of this pathway in CCM development, progression and gene dysregulation following differential editing of miRNAs and may be a valid field for new findings. The role of this class of regulatory RNAs, indeed, is still poorly investigated in CCM and only two miRNAs, miR-27a and mmu-miR-3472a, seem to be upregulated, and six, miR-125a, miR-361-5p, miR-370-3p, miR-181a-2-3p, miR-95-3p, and let-7b-3p, downregulated in CCM endothelial cells [63]. Of these, miR-27a, miR-let-7b, miR-let-7bHG and miR-181A2HG were differentially edited in our CCM-ECs. Briefly, miR-27a negatively regulates VE-cadherin and was shown to be upregulated in CCM endothelial cells, suggesting that this mechanism can contribute to the onset of the pathological phenotype [64]. In contrast, the role of miR-let-7b and miR-181a-2 in CCM pathogenesis has not yet been clarified. However, the main accreditable hypothesis is that they act by targeting genes involved in angiogenesis [65]. Finally, a study conducted by Orso et al. demonstrated that miR-21targets *KRIT1* mRNA 3ʹUTR inducing its down-expression [66,67]. Also, miR-21 was differentially edited in our samples. Our pool of differentially edited miRNAs comprises 207 transcripts targeting 757 genes involved in pathways related to CCM onset, suggesting that this pool may comprise valid miRNAs for further investigation for the development of CCM targeted therapy. Although the hypothesis is that editing modifications occurring in miRNAs and other regulatory RNAs can interfere with target binding, the role of editing modifications on miRNAs biological activity remains to be demonstrated.

### Limitations of the study

This study represents the first description of the editome profile of endothelial cells isolated from sporadic CCM biopsies. Although important findings can be further investigated, some limitations have to be mentioned: the reduced sample number due to the low number of patients who undergo surgery, and the difference of the quantity of outputted data between the two samples. The same RNA concentration was used to generate cDNA libraries, however, a huge, different, number of reads were given by the sequencing run. Moreover, another critical point is the possibility that some variants detected in RNA molecules may occur following somatic mutations. However, also in this case, it is possible that somatic variants can affect RNA physiological activity.

## Conclusions

In order to map the editing profile of CCM-ECs, a comparison between the editomes of HBMECs and CCM-ECs allowed to identify a huge number of both coding and noncoding transcripts, which underwent differential editing events. Some editing modifications were limited to HBMECs suggesting that they may be particular to BBB endothelial cells and essential for maintaining its properties. In contrast, it was observed that differentially edited genes take part in pathway and signalling cascades that are known to be involved in CCM pathogenesis. Well-matched results were obtained by clustering analysis of differentially edited miRNAs. Interestingly, two novel pathways are worthy of mention and these are the PAR1-mediated thrombin cascade and the noncanonical Wnt signalling, suggesting that elucidating mechanisms of CCM that have not yet been well clarified, in particular, for the sporadic form of the disease, might be considered. Moreover, a remarkable number of differentially edited miRNAs were differentially edited in CCM-ECs giving a new source for investigations aimed to develop targeted therapies.

## Supplementary Material

Supplemental Material

## Accession to the raw data

Raw data are accessible at the Sequence Read Archive (SRA), BioProject ID: PRJNA656789.
