# Supplementary material for: Editome landscape of CCM-derived endothelial cells
Source: RNA Biol. 2022 Jun 30;19(1):852–65. doi: 10.1080/15476286.2022.2091306 (PMC9248949; doi:10.1080/15476286.2022.2091306)
Supplement: Supplemental Material [file KRNB_A_2091306_SM7022.zip › Supplementary Materials captions.docx]

**Supplementary Materials captions**

**SM1: RNA-sequencing statistics.** The table summarizes statistics of each RNA sequencing run. As shown, each sample was sequenced in triple.

**SM2: REDItools output.** The file lists annotated editing sites detected in HBMECs, CCM-ECs1, CCM-ECs2 (sheets 1 to 3, respectively) and the *de-novo* ones (sheets 4 to 6, in the same order).

**SM3: InteractiVenn output.** The two files (SM3a and SM3b) include the gene lists outputted by the InteractiVenn tool. SM3a refers to the annotated editing sites. SM3b refers to the *de-novo* ones.

**SM4: Variant Effect Predictor (VEP) output_Annotated sites.** The tables list annotation results obtained by the VEP tool of the Ensembl Genome browser for the annotated editing sites detected by the REDItools. For each sample, input files were divided according to the enzymatic modification. The file is organized as follow: HBMECs A-to-I, miscellaneous modifications (files 4); CCM-ECs1 A-to-I, miscellaneous modifications (files4b-4c); CCM-ECs2 A-to-I, miscellaneous modifications (file 4c).

**SM5: Variant Effect Predictor (VEP) output_DeNovo sites.** The tables list annotation results obtained by the VEP tool of the Ensembl Genome browser for the *de-novo* editing sites detected by the REDItools. For each sample, input files were divided according to the enzymatic modification. The file is organized as follow: HBMECs A-to-I, C-to-U, miscellaneous modifications (Sheets 1-3); CCM-ECs1 A-to-I, C-to-U, miscellaneous modifications (Sheets 4-6); CCM-ECs2 A-to-I, C-to-U, miscellaneous modifications (Sheets 7-9).

**SM6: Figure 4 gene lists.** The tables list genes as distributed in the Venn diagrams of the Figure 4. Each sheet is named with the same letter of the Venn diagram it refers.

**SM7: Editing ratio values.** The file comprises 24 sheets, one for each chromosome (22 autosomes, X chromosome, mitochondrial chromosome). In each sheet, editing sites are listed according to their position on the chromosome. Editing ratio (ER) was calculated by dividing frequency of the editing event between sample and control. ER=0: editing site only detected in HBMECs; ER<1: editing event less frequent in the sample; ER=1: no differences in editing frequency between sample and control; ER>1: higher frequency of the editing event in the sample. **De-novo* editing modification.

**SM8: FunRich outputs.** SM8 comprises 4 files (a, b, c, d) containing output information of the enrichment analysis performed by the FunRich tool. Each file (a-c) is made by 10 sheets reporting the gene dataset, results for gene clustering according to the GeneOntology annotation terms (sheets 2-4), the Reactome biological pathways (sheet 5), protein domain (sheet 6), site of expression (sheet 7), transcription factors (sheet 8), clinical phenotype (sheet 9) and somatic mutations (sheet 10). SM8a: output of the first cluster; SM8b: output of the second cluster; SM8c: output of the third cluster. SM8d: output of miRNA enrichment. The file comprises 3 sheets in which fold enrichment, percentage of genes and biological pathways are reported.
